# Association between non-compliance with psychiatric treatment and non-psychiatric service utilization and costs in patients with schizophrenia and related disorders

**DOI:** 10.1186/s12888-016-1156-3

**Published:** 2016-12-12

**Authors:** Soohyun Joe, Jung Sun Lee

**Affiliations:** 1 Department of Psychiatric rehabilitation, Bugok National Hospital, Changnyung, Kyungnam Republic of Korea; 2 Department of Psychiatry, University of Ulsan College of Medicine, Asan Medical Center, 88, Olympic-ro 43-Gil, SongPa-Gu, Seoul, 05505 Republic of Korea

**Keywords:** Medical costs, Medication adherence, Medication compliance, Medication persistence, Psychiatry, Service costs

## Abstract

**Background:**

The relationship between medication non-compliance in patients with schizophrenia and related disorders, and increased psychiatric service utilization and costs are well documented; however, non-psychiatric service utilization and costs are not. Therefore, we investigated the association of non-compliance with psychiatric treatment and the utilization and costs of non-psychiatric services.

**Methods:**

Data on South Korean individuals with a lifetime diagnosis of schizophrenia or a related disorder, who were treated in a psychiatric clinic at least twice during 2011, were selected among national data collected for the Health Insurance Review and Assessment Service-National Patients Sample between January 1, 2011 and December 31, 2011. The sample was divided into two overlapping groups with two different classifications of patterns of medication prescription refills: (1) adherent versus non-adherent group, and (2) persistent versus non-persistent group. A matching method was used to remove the effects of different follow-up durations and insurance system on medical service utilization and costs. The final sample for analysis consisted of data from 5,548 individuals in the adherent versus non-adherent group and 3,912 in the persistent versus non-persistent group. Comparisons of the psychiatric and non-psychiatric service utilizations were made between the groups.

**Results:**

The number of psychiatric service utilizations were significantly lower in the non-adherent than the adherent group. They were also significantly lower in the non-persistent group than the persistent group. The number of non-psychiatric service utilizations was significantly higher in the non-adherent group. They were also significantly higher in the non-persistent group than the persistent group. All psychiatric costs per person during the study period were lower in the non-adherent than the adherent group, and lower in the non-persistent than the persistent group. All non-psychiatric costs per person were higher in the non-adherent than the adherent group, and higher in the non-persistent than the persistent group.

**Conclusion:**

Non-adherence to psychiatric treatment by patients with schizophrenia and related disorders was associated with higher medical service utilization and increased personal and societal medical costs.

**Electronic supplementary material:**

The online version of this article (doi:10.1186/s12888-016-1156-3) contains supplementary material, which is available to authorized users.

## Background

Poor medication adherence is common among patients with schizophrenia, and is associated with higher healthcare resource utilization and costs [1–5]. Medication non-compliance, which is related to poor psychosocial functioning [6–8], poor self-care, and an unhealthy lifestyle, increases the probability of medical service utilization and costs. It also may negatively affect non-psychiatric service utilization and costs. Although research has examined the association of medication non-compliance with healthcare service utilization and costs [3, 6, 9–12], most studies have focused on psychiatric services, such as psychiatric hospitalization and community day-programs. Little attention has been given to non-psychiatric service utilization and costs. The relationship between non-compliance and the use of psychiatric services varies across types of services [5, 10, 13, 14]. For instance, non-compliance is associated with the use of medical clinic services [15], which might be because non-compliant patients exhibit fewer help-seeking behaviors. Whether non-compliance with psychiatric medications by patients with schizophrenia affects non-psychiatric service utilization and costs has not been investigated. Therefore, the impact of psychiatric medication compliance on non-psychiatric service utilization and costs should be evaluated.

A previous study on psychiatric and non-psychiatric service utilization comparing patients continuing antipsychotics and patients discontinuing them [15], found that the patients continuing antipsychotics had higher rates of emergency-room use, out-patient hospitalizations, and other out-patient services for both psychiatric and non-psychiatric reasons. However, the study did not investigate the association of adherence (i.e., the extent to which the patient took the medication as prescribed) with psychiatric and non-psychiatric service utilization. In addition, previous studies have not measured the costs of psychiatric and non-psychiatric service utilization. A study of the impact of compliance with psychiatric and non-psychiatric service use, using systematic measures of compliance and healthcare costs, was needed. Hence, this study investigated the associations between psychiatric medication non-compliance and psychiatric and non-psychiatric service utilization and costs separately, using two dimensions of compliance: adherence and persistence.

## Methods

### Data characteristics

This study analyzed data from the Health Insurance Review and Assessment Service-National Patients Sample (HIRA-NPS) between January 1, 2011 and December 31, 2011 (NPS-2011-0101). The HIRA-NPS is a stratified, random sample from a South Korean health insurance review database. Detailed information about the HIRA-NPS is provided in Additional file 1. As this information is public data and was unlinked to personal identifiers, informed consent was not necessary and unobtainable. This study was approved by the institutional review board of Ulsan University Hospital (IRB File No: 2015-10-020) with which the first author is affiliated.

### Selection of patient data

The patient selection process is illustrated in Additional file 2. Patients who met the following criterion were included: a diagnosis of schizophrenia or related disorder as defined by the 6^th^ revision of the Korean Standard Classification of Diseases [16], which is a modification of the International Classification of Diseases-10 [17]. Patients who met the following criteria were excluded: (a) a diagnosis of schizophrenia or related disorder treated only in a non-psychiatric clinic (e.g., oriental medicine or public health) during the follow-up period and (b) patients with follow-up periods that were too short to measure compliance (i.e., the first visit was during the follow-up period, but the second one was scheduled after the follow-up period).

### Psychiatric medications

The psychiatric medications identified as those used by the patients in our sample included: (1) antipsychotics: amisulpride, aripiprazole, haloperidol, clozapine, olanzapine, paliperidone, risperidone, quetiapine, ziprasidone, chlorpromazine, sulpiride, zotepine, blonanserin, pimozide; (2) mood stabilizers: lithium, valproate, carbamazepine, lamotrigine; (3) antidepressants: amitriptyline, imipramine, tianeptine, mirtazapine, trazodone, escitalopram, fluoxetine, fluvoxamine, paroxetine, sertraline, duloxetine, milnacipran, velnafaxine, moclobemide, naltrexone, acamprosate, bupropion, nortryptiline, clomipramine; (4) anti-anxiety drugs: diazepam, clorazepam, chlordiazepoxide, buspirone, alprazolam, flunitrazepam, clonazepam, clobazam and (5) sleeping pills: zolpidem. Only the oral form of the medication was used to measure compliance because compliance with depot medication is distinct from the oral medication and the proportion of unopposed usage of depot form is smaller than the oral form.

### Compliance measures

We measured two dimensions of compliance and classified the patients into the two dimensions: adherence and persistence [18]. Adherence usually refers to the extent to which a patient takes medicine as prescribed, whereas persistence refers to the continuation with the medication as prescribed. This differentiation is clinically meaningful because non-adherent behavior and non-persistent behavior may have different social and psychological causes, which can be solved by different intervention strategies [19]. Many non-persistent patients may be non-adherent at the same time. However, the dimensions are not the exactly same. Non-adherent patients are not always non-persistent and vice versa.

In this study, we defined adherence as the extent to which a patient was prescribed psychiatric medication between the first psychiatric visit and the last psychiatric visit during 2011. Duration of follow-up was defined as number of days between the first psychiatric visit and the last psychiatric visit during 2011. We excluded the last prescription because the patients might not have taken their last prescribed medicine when they were lost to follow-up, and because the last prescription might have been finished after the end of the follow-up period. Persistence in this study was defined as whether a psychiatric prescription was interrupted during the follow-up period.

#### Adherence patterns

Adherence to psychiatric treatment was evaluated by calculating the percentage of days of psychiatric prescription (PDP): PDP = (Total days of the psychiatric prescription - Number of prescription days to the last day of the prescription - Total days of overlapping prescription (s) due to a premature visit)/(Duration of follow up, i.e., number of days between the first psychiatric visit and the last psychiatric visit). We defined adherence as a PDP ≥ 80% and non-adherence as a PDP < 80%.

#### Persistence patterns

The interruption of continuous psychiatric medication was defined as having a refill gap of 8 weeks or longer and a re-initiation of medication during the follow-up period. Patients with at least one or more interruptions were classified as non-persistent, and patients without an interruption were classified as persistent.

### Matching procedure

Data for the final analysis were obtained by matching the duration of follow up and the distribution of the types of insurance between the adherent and non-adherent groups, and between the persistent and non-persistent groups. A total of 7,848 patients met the inclusion criteria. The mean duration of follow up was 319.97 days (SD = 72.98) and the mean duration of psychiatric prescriptions was 213.23 days (SD = 130.26). We divided the total sample into adherent and non-adherent groups, and matched the duration of follow up and insurance type between the groups using a propensity-score matching method. Each of the 2,774 non-adherent patients was matched with a patient in the corresponding group. We also divided the total sample into a persistent group, in which patients continued their medication (medication without interruption ≤ 56 days) and a non-persistent group, in which patients discontinued their medication at least once (medication with interruption > 56 days), and matched the duration of follow-up and insurance type of these two groups. Each of the 1,956 non-persistent patients was matched with a patient in the corresponding group. We identified 1,041 patients who were both non-adherent and non-persistent in the non-adherent and non-persistent groups. In the non-persistent group, 915 patients (62.5%) were non-persistent but adherent; and in the non-adherent group, 1,733 patients were non-adherent (46.8%) but persistent.

### Medical service utilizations and costs

The number of visits to psychiatric clinics, number of psychiatric hospitalizations, duration of psychiatric hospitalizations, duration of psychiatric medications, number of visits to medical clinics, number of medical hospitalizations, duration of medical hospitalizations, duration of psychiatric medications, number of emergency room visits, number of deaths in the follow-up period, number of surgeries, and the medical costs during the year 2011, were investigated.

### Statistical analyses

All statistical analyses were conducted using the STATA (Version 13, STATA Corp, LP) statistical package. Group comparisons were made with chi-square tests for categorical variables and Student’s t-tests for continuous variables. Poisson regression, multiple regression, and logistic regression analysis, adjusted for sex, age, and follow-up duration were used to determine the association between non-compliance (non-adherent and non-persistent) and the frequency of using various healthcare services and mortality. The original data did not provide information about the severity of illness of the individuals in the sample. Therefore, we used the average daily chlorpromazine equivalent dose of antipsychotics as an indirect measure to control for the severity of illness. We assumed that the prescription of a higher antipsychotic dose would reflect a greater severity of mental illness. The daily chlorpromazine equivalent dose of antipsychotics was calculated using several published equivalencies for antipsychotics [20–24].

## Results

### Demographic and clinical characteristics

The mean age of the 5,548 patients in the adherent and non-adherent groups was 48.28 years (SD = 16.91) and that of the 3,912 patients in the persistent and non-persistent groups was 49.87 years (SD = 18.73). Schizophrenia was the most common diagnosis (75.34 and 69.81%) for the adherence/non-adherence and persistent/non-persistent classifications, respectively). The mean duration of follow up was 332.54 days (SD = 58.17) for the adherence/non-adherence classification and 316.98 days (SD = 68.45) for the persistent/non-persistent classification. The mean duration of psychiatric medication treatment was 218.62 days (SD = 125.93) for the adherence/non-adherence classification and 158.12 days (SD = 132.84) for the persistent/non-persistent classification.

There was no statistically significant difference in gender between the adherent and non-adherent groups (*p* = 0.7680) or the persistent and non-persistent groups (*p* = 0.8230). However, the mean age was significantly higher (*p* < 0.001) in the non-adherent (50.71, [SD = 18.58]) than the adherent group (45.85, [SD = 14.66], *p* < 0.001) and in the non-persistent (54.16, [SD = 20.67]) than the persistent group (45.59, [SD = 15.42]). The distribution of diagnoses was significantly different between the adherent and non-adherent groups and the persistent and non-persistent groups. The proportion of diagnoses of schizophrenia among all diagnoses of schizophrenia and related disorders was larger in the adherent group (79.2%) than the non-adherent group (71.5%). The proportion of diagnoses of schizophrenia among all diagnoses of schizophrenia and related disorders was larger in the persistent group (74.3%) than the non-persistent group (65.3%). The matching procedure eliminated potential differences in follow-up duration and insurance type between the groups. The mean duration of psychiatric medications was significantly lower in the non-adherent (133.77, [SD = 117.53]) than the adherent group (303.48, [SD = 59.21]) and lower in the non-persistent (51.22, [SD = 73.83]) than the persistent group (265.02, [SD = 83.56]).

### Adjusted influence of non-compliance (non-adherence and non-persistence) on psychiatric and non-psychiatric service utilization

Table 1 presents the utilization of psychiatric and non-psychiatric services by compliance (adherence and persistence) pattern. With respect to the influence of non-adherence (versus adherence) on the utilization of psychiatric services, the total number of visits to psychiatric clinics was significantly lower in the non-adherent group by 0.469 (95% CI: −0.487–0.450, *p* < 0.001). The total number of psychiatric hospitalizations was significantly lower in the non-adherent group by 0.049 (95% CI: −0.086–-0.012, *p* = 0.009). The total duration of psychiatric hospitalizations was not significant (*p* = 0.309), and the duration of psychiatric medications was significantly lower in the non-adherent group by 138.05 days (95% CI: −142.893–-133.210, *p* < 0.001).

**Table 1:** Utilization of psychiatric and non-psychiatric services by adherence and persistence patterns

	Non-adherent (*N* = 2774)		Adherent (*N* = 2774)		B	*p*	Non-persistent (*N* = 1956)		Persistent (*N* = 1956)		B	*p*
	Mean	SD	Mean	SD			Mean	SD	Mean	SD		
Psychiatric-related service utilization												
Total number of visits to psychiatric clinics^1)^	6.51	6.38	10.89	8.30	−0.469	<0.001	5.03	5.85	10.14	7.84	−4.543	<0.001
Total number of psychiatric hospitalizations^1)^	2.02	3.95	2.15	4.42	−0.049	0.009	0.97	2.57	1.76	3.86	−0.749	<0.001
Total duration of psychiatric hospitalizations^2)^ (days)	56.60	202.61	63.61	209.34	−5.592	0.309	24.34	69.94	57.81	305.52	−32.438	<0.001
Total duration of psychiatric medications^2)^ (days)	210.42	131.03	354.41	66.26	−138.052	<0.001	146.36	136.95	314.34	95.96	−166.17	<0.001
Non-psychiatric related service utilization												
Total number of visits to medical clinics^1)^	4.62	7.34	2.62	6.98	0.354	<0.001	6.02	8.17	3.21	7.08	2.132	<0.001
Total number of medical hospitalizations^1)^	1.04	2.59	0.26	1.25	1.136	<0.001	1.34	2.81	0.36	1.36	0.760	<0.001
Total duration of medical hospitalization^2)^ (days)	50.08	433.04	5.63	31.59	39.530	<0.001	72.70	587.91	13.82	209.13	47.869	0.001
Total duration of medical medications^2)^ (days)	131.75	178.52	64.87	147.37	50.437	<0.001	174.92	190.56	81.50	162.69	66.652	<0.001
Total number of emergency room visits^1)^	0.31	1.30	0.14	1.08	0.737	<0.001	0.32	1.04	0.17	1.04	0.106	0.002
Total number of surgeries^1)^	0.13	0.48	0.02	0.17	1.442	<0.001	0.19	0.59	0.04	0.25	0.115	<0.001
					OR	P					OR	P
Number of deaths during the follow-up period^3)^	0.03	0.17	0.02	0.14	1.199	0.324	0.03	0.18	0.01	0.11	1.950	0.007

**p < 0.001; adherence is defined as the percentage of days of psychiatric prescription (PDP) ≥ 80% and non-adherence as a PDP < 80%; participants consisted of patients with schizophrenia and related disorders; ^1)^ results were obtained by Poisson regression analysis with the general linear model; ^2)^ results were obtained by multiple linear regression analysis; and ^3)^ results were obtained by logistic regression analysis

The number of visits to medical clinics significantly increased by 0.354 (95% CI: 0.325–0.384, *p* < 0.0001), the number of medical hospitalizations significantly increased by 1.136 (95% CI: 1.052–1.220, *p* < 0.001), and the duration of medical hospitalizations significantly increased by 39.53 days (95% CI: 23.199–55.861, *p* < 0.001) in the non-adherent group. In addition, the total duration of medical medication significantly increased by 50.44 days (95% CI: 42.447–58.427, *p* < 0.001), the number of emergency room visits significantly increased by 0.737 (95% CI: 0.615–0.859, *p* < 0.0001), and the number of surgeries significantly increased by 1.442 (95% CI: 1.172–1.711, *p* < 0.0001,) in the non-adherent group. The mortality rate was not significantly (*p* = 0.324) different between non-adherent and adherent group.

The results of the influence of non-persistence (versus persistence) on the utilization of psychiatric services showed that the total number of visits to psychiatric clinics was significantly lower by 4.543 (95% CI: −4.966–4.121, *p* < 0.001) and the number of psychiatric hospitalizations was significantly lower by 0.749 (95% CI: −0.953–0.545, *p* < 0.001) in the non-persistent group. The total duration of psychiatric hospitalizations was significantly lower by 32.44 days (95% CI: −46.661–-18.214, *p* < 0.001) and the duration of psychiatric medications was significantly lower by 160.17 days (95% CI: −166.650–-153.693, *p* < 0.0001) in the non-persistent group.

Non-persistence influenced the utilization of non-psychiatric services, significantly increasing the total number of visits to medical clinics by 2.132 (95% CI: 1.657–2.606, *p* < 0.0001), the number of medical hospitalizations by 0.760 (95% CI: 0.622–0.898, *p* < 0.0001), and duration of medical hospitalizations by 47.87 days (95% CI: 19.420–76.317, *p* < 0.001) in the non-persistent group. Non-persistence significantly increased the total duration of medical medications by 66.65 days (95% CI 56.374–76.930, *p* < 0.0001), the number of emergency room visits by 0.106 (95% CI: 0.039–0.172, *p* < 0.002), and the number of surgeries by 0.115 (95% CI: 0.086–0.144, *p* < 0.0001) in the non-persistent group. The mortality rate was significantly higher in the non-persistent than the persistent group (OR, 1.950, 95% CI: 1.195–3.181, *p* < 0.007).

### Adjusted influence of non-compliance (non-adherence and non-persistence) on psychiatric and non-psychiatric costs

Table 2 presents the yearly medical service costs per person with schizophrenia and related disorders (1,000 Korean Won was equivalent to 1.1–1.2 US dollars in 2011). After adjusting for the covariates i.e., sex, age, duration of follow-up, and insurance type (national health insurance or others), all of the associations between the compliance patterns and non-psychiatric costs were statistically significant. The total psychiatric costs were significantly lower by 783,300 won (*p* < 0.001) in the non-adherent group. Psychiatric copayments and government payments were also lower by 74,800 and 70,500 won respectively (*p* ≤ 0.001), in the non-adherent group. The total non-psychiatric costs were significantly higher by 1,401,300 won (*p* < 0.001) in the non-adherent group. Non-psychiatric copayments and government payments were also higher by 163,900 and 1,219,700 won respectively (*p* < 0.001), in the non-adherent group. The combined psychiatric and non-psychiatric total costs were higher by 618,000 won in the non-adherent group (*p* = 0.001). The combined psychiatric and non-psychiatric copayments and the government payments were also higher by 89,100 and 514,600 won respectively, in the non-adherent group.

**Table 2:** Psychiatric and non-psychiatric costs by adherence and persistence patterns

	Non-adherent (*N* = 2774)		Adherent (*N* = 2774)		*B*	*p*	Non-persistent (*N* = 1956)		Persistent (*N* = 1956)		*B*	*p*
	Mean	SD	Mean	SD			Mean	SD	Mean	SD		
Psychiatric-related costs												
Total costs	3061	5101	3907	5954	−783.3	<0.001	1553	3170	3317	5341	−974.8	<0.001
Copayments	343	826	420	973	−74.8	0.001	251	617	445	938	−69.4	<0.001
Government payments	2697	4585	3463	5307	−705.0	<0.001	1293	2759	2845	4632	−897.7	<0.001
Non-psychiatric related												
Total costs	1900	6527	211	1450	1401.3	<0.001	3039	8532	327	1673	1614.1	<0.001
Copayments	243	988	28	259	163.9	<0.001	397	1238	52	291	200.1	<0.001
Government payments	1628	5715	181	1220	1219.7	<0.001	2605	7538	266	1373	1403.0	<0.001
Total psychiatric and non-psychiatric-related costs												
Total costs	4961	7867	4118	6109	617.996	0.0010	4592	8803	3644	5626	639.302	0.0100
Copayments	586	1261	448	1009	89.090	0.0020	648	1355	497	1003	717.831	0.0010
Government payments	4324	7004	3644	5434	514.649	0.0020	3899	7801	3112	4863	505.311	0.0190

**p < 0.001; adherence is defined as the percentage of days of psychiatric prescription (PDP) ≥ 80% and non-adherence as a PDP < 80%; currency unit = 1,000 Korean Won; data were collected from patients with schizophrenia and related disorders; the covariates included sex, age, duration of follow up, and insurance type (national health insurance or another type)

The total psychiatric costs were significantly lower by 974,800 won (*p* = 0.001) in the non-persistent group. The psychiatric copayments and government payments were also lower by 69,400 and 897,700 won respectively (*p* < 0.01), in the non-persistent group. The total non-psychiatric costs were significantly higher by 1614,100 won (*p* < 0.001) in the non-persistent group. The non-psychiatric copayments and government payments were also higher by 200,100 and 1403,000 won respectively (*p* < 0.001), in the non-persistent group. The combined psychiatric and non-psychiatric total costs were higher by 639,302 won in the non-persistent group (*p* = 0.01). The combined psychiatric and non-psychiatric copayments and government payments were also higher by 717,831 and 505,311 won respectively (*p* < 0.05), in the non-persistent group.

## Discussion

This is the first study to compare psychiatric and non-psychiatric service utilization and costs between groups by evaluating two different compliance patterns. It is also the first study to investigate compliance, medical service utilization, and personal and societal costs among patients with schizophrenia and related disorders from a nationwide survey of an East Asian country.

The lower service utilization (i.e., psychiatric visits and total prescriptions) by patients who were non-compliant in this study might be a direct result of lower compliance itself because non-compliant patients tend to avoid using healthcare services. However, the duration of psychiatric hospitalization was not significantly different between the non-adherent and the adherent groups. This might be due to the higher relapse frequency in the non-adherent group, also documented in other studies [1, 2, 25]. In those studies, non-adherent patients tended to refuse hospitalization although frequent exacerbations of psychosis increased the likelihood of hospitalization. In this study, the higher number of relapses and hospitalizations might have compensated for the lower service utilization associated with non-adherence. The shorter duration of hospitalization of non-persistent patients might be related to the shorter duration of the follow-up period. Long-term follow-up might have led to shorter hospitalizations of the non-adherent than the adherent group. Another possibility is that the length of hospitalization might be more variable for those with the adherence rather than the persistence pattern. Non-persistent behavior, characterized by interruptions in treatment, might have a stronger relationship with premature discharge than non-adherent behavior.

In contrast, medical service utilization was higher in the non-compliant groups, suggesting that it was related to a higher number of medical comorbidities. Medical comorbidities among patients visiting psychiatric clinics have been estimated at 43–47% [26–28], and found to be responsible for 60% of premature deaths in patients with schizophrenia, excluding suicide [29].

These findings might be related to higher rates of unhealthy lifestyle behaviors, such as smoking, excessive alcohol consumption, and poor self-care habits (e.g., hygiene, diet, and exercise) in patients with schizophrenia [4]. Patients with schizophrenia receive little medical attention from physicians [30], and therefore, may have fewer medical visits and documented medical problems. Thus, higher medical service utilization in non-compliant patients suggests that non-compliance might be associated with medical comorbidities that are more severe. Unhealthy lifestyle habits in patients with a severe mental illness, who are non-compliant, may result in poor physical health and higher utilization of medical services. Non-compliance might be another indicator of the illness’ severity and result in higher mortality and medical morbidity.

Our results suggest that non-compliance was related to higher health service costs and may be related to higher medical service utilization by non-compliant patients. Despite lower psychiatric service utilization by the non-compliant patients, their total healthcare costs were higher than those in the compliant group, suggesting that non-compliance with psychiatric prescriptions influenced total healthcare costs by increasing medical healthcare costs. Increased medical costs were sufficiently higher to reverse the effect of the decrease in psychiatric costs of the non-compliant group. Both personal and governmental burdens, as measured by the medical and total health care costs were higher in the non-compliant group. Thus, there is a pressing need to treat patients with schizophrenia and related disorders and for government to support this effort. Increasing the compliance of patients with these diagnoses may directly reduce the national economic burden.

Among the compliance dimensions, persistence had a strong association with increased utilization of non-psychiatric services. This suggests that the discontinuation of psychiatric treatment or a long interval between psychiatric treatments might play a more significant role in maintaining the medical health of this patient population than the percentage of medications taken during the follow-up period. Alternatively, persistence rather than adherence might have a greater influence on medical service utilization. Therefore, non-persistent patients might be more non-compliant than non-adherent patients, and they might be more likely to neglect the self-care activities needed to stay healthy. Both of the compliance dimensions had similar influences on medical service costs, indicating that unhealthy behaviors had a similar influence on the economic aspects of healthcare utilization. The differences in cost per visit between the non-persistent and persistent patients may be greater than that between the non-adherent and adherent patients.

This study has several limitations, all of them related to the limitations of the original data. First, the follow-up period was relatively short. One year was the maximum follow-up period because new samples for the national data set are selected yearly. Second, the original data did not include information about additional out-of-pocket payments by patients. Although budgeting for out of pocket payments is strictly limited by the South Korean government, one should remember that actual healthcare service costs are larger than those cited in this study are. Third, the analyses of data from this sample might not be representative because the participants were selected from a subset of patients with schizophrenia and related disorders, and matched on their insurance type and availability for the follow-up period. Fourth, baseline psychiatric characteristics, such as age at onset, duration of illness, previous number of hospitalizations, and duration of psychiatric treatment were not analyzed because past psychiatric history was not available in the original data. Therefore, the baseline data of the sample were not the same although the representativeness of the national sample and the adequate sample size might compensate for this limitation.

## Conclusion

Non-compliance of psychiatric treatment in persons with schizophrenia and related disorders is associated with higher medical service utilization and increased medical costs.

## Additional files

Additional file 1: Characteristics of the data from the Health Insurance Review and Assessment Service-National Patients Sample (HIRA-NPS). (DOCX 18 kb)
Additional file 2: The patient selection process. The arrows between the boxes depict ‘the next phase of the selection process’. SCZ, Schizophrenia and its related disorders; PDP, Percentage of days of psychiatric prescription. (DOCX 44 kb)

## Abbreviations

HIRA-NPS: Health Insurance Review and Assessment Service-National Patients Sample
PDP: Percentage of days of psychiatric prescription
