# Supplementary material for: Association between non-compliance with psychiatric treatment and non-psychiatric service utilization and costs in patients with schizophrenia and related disorders
Source: BMC Psychiatry. 2016 Dec 12;16:444. doi: 10.1186/s12888-016-1156-3 (PMC5154112; doi:10.1186/s12888-016-1156-3)
Supplement: Additional file 1: — Characteristics of the data from the Health Insurance Review and Assessment Service-National Patients Sample (HIRA-NPS). (DOCX 18 kb) [file 12888_2016_1156_MOESM1_ESM.docx]

Characteristics of the data from the Health Insurance Review and Assessment Service-National Patients Sample (HIRA-NPS)

The Health Insurance Review and Assessment Service-National Patients Sample (HIRA-NPS), which consists of health insurance data from South Korea’s population, is an age- and sex-stratified, random sample from the Korean National Health Insurance Review and Assessment database. Multistage cluster sampling was used to select the samples [1]. Based on the 2010 census data, the nation was divided into 12 areas, and 246 sections were selected from each area. All the families were included from each section, and one individual between 18 and 74 years was randomly selected from each family. Approximately 3% of the population was selected to be part of the HIRA-NPS, which consists of 2011 census data on 1,375,842 patients, and includes information related to demographic characteristics, diagnosis for each visit, medications, and healthcare-service utilization records for each visit to a hospital, private clinic, public health center, oriental medical clinic, dental clinic, and midwife center. All personal identifiers were removed and unlinked from the data before they were transferred to the authors. In order to analyze medical information longitudinally, an identification number was assigned to each patient.

1. Nayak RK, Doose DR, Nair NP. The bioavailability and pharmacokinetics of oral and depot intramuscular haloperidol in schizophrenic patients. J Clin Pharmacol. 1987;27:144-150.
