# Supplementary material for: Association between non-compliance with psychiatric treatment and non-psychiatric service utilization and costs in patients with schizophrenia and related disorders
Source: BMC Psychiatry. 2016 Dec 12;16:444. doi: 10.1186/s12888-016-1156-3 (PMC5154112; doi:10.1186/s12888-016-1156-3)
Supplement: Additional file 2: — The patient selection process. The arrows between the boxes depict ‘the next phase of the selection process’. SCZ, Schizophrenia and its related disorders; PDP, Percentage of days of psychiatric prescription. (DOCX 44 kb) [file 12888_2016_1156_MOESM2_ESM.docx]

Diagnosed with SCZ at least once during lifetime

N = 8276

Exclusion: Patients treated only in a non-psychiatric clinic

N = 8042

Exclusion: Patients with follow-up periods that were too short to measure compliance

N = 7848

Matching duration of follow-up and distribution of insurance types by adherence pattern

N = 5548

Matching duration of follow-up and distribution of insurance types by persistence pattern

N = 3912

Non-persistent group

Number of interruptions ≥ 1

N = 1956

Persistent group

Number of interruptions ≥1

N = 1956

Non-adherent group: PDP < 80

N = 2774

Adherent group: PDP ≥ 80

N = 2774

Additional file 2. The patient selection process. The arrows between the boxes depict ‘the next phase of the selection process’. SCZ, Schizophrenia and its related disorders; PDP, Percentage of days of psychiatric prescription.
